# Expression, Localization, and Protein Interactions of the Partitioning Proteins in the Gonococcal Type IV Secretion System

**DOI:** 10.3389/fmicb.2021.784483

**Published:** 2021-12-16

**Authors:** Melanie M. Callaghan, Birgit Koch, Kathleen T. Hackett, Amy K. Klimowicz, Ryan E. Schaub, Natalio Krasnogor, Joseph P. Dillard

**Affiliations:** ^1^Department of Medical Microbiology and Immunology, University of Wisconsin-Madison, Madison, WI, United States; ^2^Interdisciplinary Computing and Complex BioSystems (ICOS), Newcastle University, Newcastle upon Tyne, United Kingdom

**Keywords:** *Neisseria gonorrhoeae* (GC), relaxosome, riboswitch, protein–protein interaction, subcellular loalization

## Abstract

Partitioning proteins are well studied as molecular organizers of chromosome and plasmid segregation during division, however little is known about the roles partitioning proteins can play within type IV secretion systems. The single-stranded DNA (ssDNA)-secreting gonococcal T4SS has two partitioning proteins, ParA and ParB. These proteins work in collaboration with the relaxase TraI as essential facilitators of type IV secretion. Bacterial two-hybrid experiments identified interactions between each partitioning protein and the relaxase. Subcellular fractionation demonstrated that ParA is found in the cellular membrane, whereas ParB is primarily in the membrane, but some of the protein is in the soluble fraction. Since TraI is known to be membrane-associated, these data suggest that the gonococcal relaxosome is a membrane-associated complex. In addition, we found that translation of ParA and ParB is controlled by an RNA switch. Different mutations within the stem-loop sequence predicted to alter folding of this RNA structure greatly increased or decreased levels of the partitioning proteins.

## Introduction

The human-restricted bacterial pathogen *Neisseria gonorrhoeae* is responsible for causing the sexually-transmitted infection gonorrhea, colonizing mucosal surfaces and causing both highly inflammatory and asymptomatic infections. In 2019, over 600,000 new cases of gonorrhea infection were reported to the Centers for Disease Control (Centers for Disease Control and Prevention, 2021); this is likely an underestimate due to the prevalence of asymptomatic infections. Antibiotic resistance in gonorrhea infections has continued to rise since the 1950s and represents an urgent worldwide health concern (Centers for Disease Control and Prevention, 2019).

A majority (60–80%) of gonococcal isolates contain the 59 kb gonococcal genetic island (GGI), which encodes a type IV secretion system (T4SS; Dillard and Seifert, 2001; Hamilton and Dillard, 2006; Shockey, 2019). The gonococcal T4SS is unique in that it secretes single-stranded DNA (ssDNA) into the extracellular space independent of cell–cell contact. Due to the natural transformability of *N. gonorrhoeae* at all stages of growth, this active DNA release can facilitate horizontal gene transfer (Dillard and Seifert, 2001; Hamilton and Dillard, 2006; Salgado-Pabón et al., 2007; Shockey, 2019). Regulation of gonococcal T4SS expression and activity is only beginning to be understood (Ramsey et al., 2015; Callaghan et al., 2021).

The GGI encodes homologues of many known T4SS proteins, providing a basis for modeling activity in this system (Hamilton et al., 2005). While many of these proteins have been further characterized, two that have not yet been addressed are the partitioning proteins, ParA and ParB (Jain et al., 2012; Kohler et al., 2013; Ramsey et al., 2014).

Partitioning proteins are found on most bacterial chromosomes and many plasmids, often as a matched pair (Bignell and Thomas, 2001). These types of proteins play a role in localizing chromosome or plasmid DNA during the process of cell division, ensuring non-random distribution of DNA molecules into daughter cells. Canonically, ParA homologues are ATPases and ParB homologues are DNA-binding proteins. Often these proteins interact with each other as a cognate pair, and ParB interacts with DNA in a sequence-specific manner (Lin and Grossman, 1998; Bignell and Thomas, 2001; Atmakuri et al., 2007).

There is limited information on the function of partitioning proteins as components of a T4SS. In the R1 plasmid conjugation system in *Escherichia coli*, ParR binds a centromere-like DNA sequence, *parC*, to facilitate the physical placement of the DNA. A recent study has shown that in this system, the association of the cognate pair of partitioning proteins ParM and ParR with the relaxase TraI, the coupling protein TraD, and the cell membrane contribute to the assembly of the apparatus and initiation of conjugative transfer (Gruber et al., 2016). In the chromosomally encoded VirB/D4 T4SS of *Agrobacterium tumefaciens*, the ParA and ParB homologues VirC1 and VirC2, respectively, interact at the cellular poles to direct relaxosome formation and DNA substrate localization. The VirC1-DNA interaction is also sequence-specific; facilitated by VirC2, VirC1 binds a DNA sequence called *overdrive* (Atmakuri et al., 2007).

**Figure 1:**
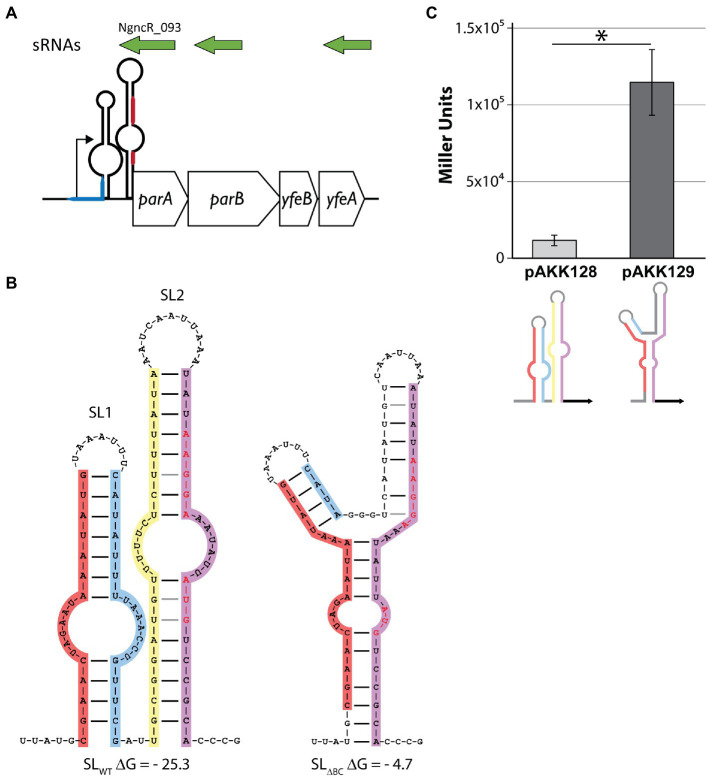
Disruption of the stem-loops in the *parA* 5′UTR increases translation of LacZ. **(A)** Schematic depicting the *parA* operon. sRNAs (green) were detected by Remmele et al. (2014). The blue line represents the *difA* site. The red line represents the Shine-Dalgarno sequence (top) and *parA* start codon (bottom). Note that *parA* 5′UTR is not to scale. **(B)** Predicted secondary structures of wild-type and mutant stem-loops. Shine-Dalgarno sequence and start codon are shown in red letters. Left: wild-type sequence. Leg A (red), leg B (blue), leg C (yellow), leg D (purple). Right: deletion of legs B and C (SL_∆BC_). **(C)** *Escherichia coli* expressing LacZ translational fusions with either the wild-type (pAKK128) or SL_ΔBC_ (pAKK129) 5′UTR*_parA_* constructs on plasmids were assayed for β-galactosidase activity. The disrupted stem-loop construct allows for >10-fold higher β-galactosidase activity, demonstrating a clear role for the native stem-loop structure in controlling protein levels. ^*^p < 0.01 by Student’s *t* test compared to SL_WT_ (*p* = 0.0012).

In the gonococcal T4SS, both *parA* and *parB* are essential for T4SS-mediated DNA secretion to occur (Hamilton et al., 2005; Pachulec et al., 2014). They are co-transcribed in an operon of the GGI distant from the other T4SS genes and near the *difA* site (Figure 1A). The *parAB* operon is transcribed at high levels compared to the rest of the characterized GGI (Pachulec et al., 2014; Ramsey et al., 2015). There is a large region of genes of unknown function between the partitioning proteins and the rest of the known T4SS protein homologues, and this region is dispensable for secretion (Pachulec et al., 2014; Callaghan et al., 2017). Little is known about the gonococcal T4SS ParAB, except that both are necessary for T4S and ParA has a conserved ATPase domain with a Walker A box that is also necessary for DNA secretion (Hamilton et al., 2005; Pachulec et al., 2014).

More is known about the regulation of T4SS expression in gonococci, and RNA-mediated mechanisms are recently emerging as the regulatory network is probed (Ramsey et al., 2015; Callaghan et al., 2021). Several sRNAs have been identified within the GGI and have yet to be functionally characterized (Remmele et al., 2014). Recent work has also implicated the Fur regulon in regulating some aspects of T4SS expression (Callaghan et al., 2021), and this regulon is known to utilize sRNA intermediates to control iron-responsive genes (Mellin et al., 2007; Yu et al., 2016). The GGI encodes an RNA switch in the *traH* 5′ untranslated region (UTR) which controls protein expression from the P*_traH_*-derived transcript (Ramsey et al., 2015). This RNA switch adopts an energetically favorable structure with two stem-loops that occludes the Shine-Dalgarno sequence and *traH* start codon. However, an alternate secondary structure becomes more energetically favorable if the upstream portion of the first stem-loop is unavailable for binding. This alternate structure is a single stem-loop that leaves the start site available for binding (Ramsey et al., 2015).

We have characterized ParAB in the gonococcal T4SS by investigating expression, protein interactions, and localization of the partitioning proteins. Our data suggest that ParAB protein expression is tightly controlled by an RNA switch. We present evidence for ParA-TraI and ParB-TraI interactions, supporting a ParAB-TraI relaxosome that initiates T4S. Finally, localization studies indicate the ParAB are unusual among partitioning proteins in that they associate with the bacterial cytoplasmic membrane.

## Materials and Methods

### Bacterial Strains and Growth Conditions

*Neisseria gonorrhoeae* MS11 and derivative strains were grown on GCB agar plates with Kellogg’s supplements or in GCBL medium with 0.042% sodium bicarbonate and Kellogg’s supplements (“cGCBL”; Kellogg et al., 1963; Morse and Bartenstein, 1974) at 37°C.

### Strain Building

Plasmids for this study (Table 1) were generated by PCR amplification of *N. gonorrhoeae* MS11 chromosomal DNA with primers listed in Table 2, followed by restriction digest with listed enzymes (Table 2). Purified, digested inserts and vectors were ligated overnight with T4 DNA ligase. Ligations were transformed in TAM1 *E. coli* (Active Motif).

**Table 1:** Plasmid constructs used in this study.

Plasmid	Description	Vector	Source/References
pMMC17	*parA*′*-FLAG3* intermediate	pMR100	This work
pMMC18	*parA*′*-FLAG3*	pMMC17	This work
pMMC20	*parB*′*-FLAG3* intermediate	pMR100	This work
pMMC21	*parB*′*-FLAG3*	pMMC20	This work
pMMC25	*NgncR_093* promoter mutant	pIDN1	This work
pMMC38	*NgncR_093* O/E (IPTG inducible) at *aspC/lctP*	pKH37	This work
pAKK128	SL_WT_-*lacZ* translational fusion	pMR115 + 1	This work
pAKK129	SL_ΔBC_*-lacZ* translational fusion	pMR115 + 1	This work
pIDN1	Cloning vector (Erm^R^)		Hamilton et al., 2001
pKH37	*cat* at *aspC/lctP*		Ramsey et al., 2012
pKH502	SL_∆BC_		This work
pMR100	FLAG3 tagging vector		Ramsey et al., 2014
**BACTH constructs**			
**Plasmid**	**Vector**	**Primer pair** ^a^	**References**
T18 TraD_N_	pUT18CT	3/4	This study
TraD_N_ T18	pUT18	3/5	This study
TraD_N_ T25	p25N	3/5	This study
T25 TraD_N_	pKT25	3/4	This study
TraI_N_ T18	pUT18	6/7	This study
TraI_N_ T25	p25N	6/7	This study
TraL_N_ T18	pUT18		Koch et al., 2020
TraL_N_ T25	p25N		Koch et al., 2020
T18 TraE_N_	pUT18C		Koch et al., 2020
T25 TraE_N_	pKT25		Koch et al., 2020
T18 TraB_N_	pUT18C		Koch et al., 2020
T25 TraB_N_	pKT25		Koch et al., 2020
T25 TraC_N_	pKT25		Koch et al., 2020
Tra_N_C T25	p25N		Koch et al., 2020
T18 TraC_N_	pUT18C		Koch et al., 2020
TraC_N_ T18	pUT18		Koch et al., 2020
TraG_N_ T25	p25N		Koch et al., 2020
TraG_N_ T18	pUT18		Koch et al., 2020
ParB_N_ T18	pUT18	49/51	This study
ParB_N_ T25	p25N	49/51	This study
T18 ParB_N_	pUT18C	49/50	This study
T25 ParB_N_	pKT25	49/50	This study
ParA_N_ T18	pUT18	52/53	This study
T25 ParA_N_	pKT25	52/53	This study
ParA_N_ T25	p25N	52/54	This study
T18 ParA_N_	pUT18C	52/54	This study
T18 TraB_F_	pUT18C		Koch et al., 2020
T25 TraB_F_	pKT25		Koch et al., 2020
T18 TraE_F_	pUT18C		Koch et al., 2020
T25 TraE_F_	pKT25		Koch et al., 2020
TraC_F_ T18	pUT18		Koch et al., 2020
TraC_F_ T25	p25N		Koch et al., 2020
T18 TraC_F_	pUT18C		Koch et al., 2020
T25 TraC_F_	pKT25		Koch et al., 2020
TraI_F_ T18	pUT18	82/84	This study
TraI_F_ T25	p25N	82/84	This study
T18 TraI_F_	pUT18C	82/83	This study
T25 TraI_F_	pKT25	82/83	This study
SopA_F_ T18	pUT18	76/78	This study
SopA_F_ T25	p25N	76/78	This study
T18 SopA_F_	pUT18C	76/77	This study
T25 SopA_F_	pKT25	76/77	This study
SopB_F_ T18	pUT18	79/81	This study
SopB_F_ T25	p25N	79/81	This study
T18 SopB_F_	pUT18C	79/80	This study
T25 SopB_F_	pKT25	79/80	This study
**BACTH vectors**	**Antibiotic resistance marker**		**Source/References**
p25N	Kan		Claessen et al., 2008
pUT18C	Amp		Karimova et al., 2001
pUT18	Amp		Karimova et al., 2001
pKT25	Kan		Karimova et al., 2001

a See Table 2, primers for BACTH constructs.

**Table 2:** Primers used in this study.

Primers			
Primer name	Sequence (5′–3′)	Assembly	Plasmid
parA_SpeIF	GTCGACTAGTATGTCCGCACCCGTAATATTG	SpeI/SmaI T4 ligation	pMMC17
parA_SmaIR2	AGTTCCCGGGTGATTGCACCTCCTTTTG		
parB_HindIIIF	CGTCAAGCTTATGAATTTGGACCAAAATAAAGC	HindIII/XhoI T4 ligation	pMMC18
parB′_XhoIR	GAGTCTCGAGGCATGGGAAAGTTTGAATGC		
parB_SpeIF	GTCGACTAGTACAGAAGAACCTGCG	SpeI/SmaI T4 ligation	pMMC20
parB_SmaIR	ATCACCCGGGCTCCTCACTCTTAGC		
parBflank_SalIF	GTGCGTCGACCTGAGCACACAGTAC	HindIII/XhoI T4 ligation	pMMC21
parBflank_XhoIR	ATGACTCGAGCTCTGAAACAGAACC		
nc093_sdmF1	GCTTTGGCAGCAGGAACTGC**G**ACG**GA**TAACAATTTACGTCTG	Site-directed mutagenesis	pMMC25
nc093_sdmR1	CAGACGTAAATTGTTA**TC**CGT**C**GCAGTTCCTGCTGCCAAAGC		
nc093F1	CATAGAGCTCGCCCCGAGAAGGAGTATCC		
nc093R1	CAGTCTCGAGCTGCATTCCCAATACATAC		
iga-end-out	ATGTGGGCGGTAAATCCTTC		
lacZ937-R	ACAGTTTCGGGTTTTCGACG		
rpoB-RT-F	TGCCGTACATGGCGGAC		
rpoB-RT-R	ATACGGGAAGGTACGCCCA		
traD-RT-F	GCGCGAAAACATGAGATTGA		
traD-RT-R	CCATGCCGATTTCCGAGTTA		
traK-RT-F	GAAGCAGCAGTATTGGCTTCGCAA		
traK-RT-R	ATTGATGCCCATATCGCCGGTAGT		
traH-RT-F	GCAATGGGAAAACTGGGTTC		
traH-RT-R	TTATCGGCTTCATGGACAAGG		
parA-RT-F	GCCTGCTTTGCCCAATTATG	Note: amplify both *parA* and *NcngR_093*	
parA-RT-R	AATTGAGGCATCGGGATACG		
parA-RT2-F	TTCCACGCAGGTTCTTCTG	Note: amplify only *parA*	
parA-RT2-R	AAGAGTCCCGGTTCATTGTC		
**Primers for BACTH constructs**			
**Primer number**	**Sequence (5′–3′)**	**Enzyme**	
3	GCTACTCTAGAGATGAGTGCCCACTTCCCTGAAAAC	XbaI	
4	CTACGGTACCCGTTAGACGGCATAACTACTTCCCTCCGT	KpnI	
5	CTACGGTACCCGGACGGCATAACTACTTCCCTCCGTA	KpnI	
6	CAAGATCTAGAGATGAAAACAAGCCTTCTCACTATTG	XbaI	
7	GCTACGAATTCGATTTTTGTTCCATTACTAATAAGTCG	EcoRI	
49	GGAAGGATCCCATGAATTTGGACCAAAATAAA	BamHI	
50	GCTACGAATTCTTACTCCTCACTCTTAGCTCC	EcoRI	
51	GCTACGAATTCGACTCCTCACTCTTAGCTCCC	EcoRI	
52	GGAAGGATCCCATGTCCGCACCCGTAATATTG	BamHI	
53	GCTACGAATTCTCATGATTGCACCTCCTTTTG	EcoRI	
54	GCTACGAATTCGATGATTGCACCTCCTTTTGCAG	EcoRI	
76	GGAAGGATCCCATGTTCAGAATGAAACTCATGGAAAC	BamHI	
77	GCTACGAATTCTTATCTAATCTCCCAGCGTGGTTT	EcoRI	
78	GCTACGAATTCGATCTAATCTCCCAGCGTGGTTT	EcoRI	
79	GGAAGGATCCCATGAAGCGTGCGCCTGTTAT	BamHI	
80	GCTACGAATTCTCAGGGTGCTGGCTTTTCAA	EcoRI	
81	GCTACGAATTCGAGGGTGCTGGCTTTTCAAGTT	EcoRI	
82	GGAAGGATCCCATGATGAGTATTGCGCAGGT	BamHI	
83	GCTACGAATTCTCAGTCTCCACCCAGG	EcoRI	
84	GCTACGAATTCGAGTCTCCACCCAGGGTT	EcoRI	

Underlined sequence indicates restriction enzyme cut site. Mutated bases are indicated in bold.

To construct pAKK128 and pAKK129, primers iga-end-out and lacZ937-R were used to PCR-amplify the *parAB* promoter region and ~1 kb of the 5′ region of *lacZ* from MMC545 (wild-type SLs) and MMC546 (SL_ΔBC_) chromosomal DNA. The PCR products were digested with ClaI (upstream of the promoter region) and XhoI (within *lacZ*), resulting in ~0.9-kb fragments that contained the *parAB* promoter with either the wild-type stem-loops or mutant stem-loops fused to the first 839 bp of *lacZ*. pMR115 + 1, which contains the full *lacZ* gene fused to a different promoter, was digested with ClaI and XhoI. The PCR products were ligated into the digested plasmid and transformed into *E. coli* TAM1, generating pAKK128 (wild-type SLs-*lacZ*) and pAKK129 (SL_ΔBC_ -*lacZ*). The constructs were confirmed by sequencing.

Plasmid pMMC25 was made using site-directed mutagenesis to alter the −10 promoter element of *NcngR_093* from TACGCT to **G**ACG**GA**: two fragments were amplified from the MS11 chromosome using primers (1) nc093_sdmF1 + nc093R1 and (2) nc093F1 + nc093_sdmR1. Base pair changes are shown in bold (Table 2). Fragments were purified and then used in equal parts as the template for a secondary PCR with primers nc093F1 + nc093R1. pIDN1 vector and purified secondary PCR product were digested with SacI/XhoI and ligated together with T4 DNA ligase before transformation into TAM1 *E. coli.*

Gonococcal strains were generated by spot transformation on GCB agar plates (Callaghan and Dillard, 2019). All strains are derived from MS11. Table 3 specifies transformations for this study as (transforming DNA) x (parent strain).

**Table 3:** Bacterial strains used in this study.

***Neisseria gonorrhoeae* strains**		
Strain name	Description	Source/References
MMC538	*parA*′*-FLAG3* pMMC18 x MS11	This work
MMC542	∆SL*-parA*′*-FLAG3* pKH502 x MMC538	This work
MMC543	∆SL-*parA*′*-FLAG3*, *cat* pKH37 x MMC542	This work
MMC544	P_Ngnc093_ -10 mutant pMMC25 x MS11	This work
MMC545	P_opaB_-SL_WT_-*lacZ* parA-lacZ WT3 gene block x MR664 (MS11 background)	This work
MMC546	P_opaB_-SL_ΔBC_-*lacZ* parA-lacZ mut2 gene block x MR661 (MS11 background)	This work
MMC547	*parB*′*-FLAG3* pMMC21 x MS11	This work
MMC548	∆SL-*parB*′*-FLAG3* pMMC21 x KH655	This work
MMC549	∆SL-*parA*′*-FLAG3, cat* pKH37 x MMC548	This work
MMC550	P_Ngnc093_ -10 mutant, *parA*′*-FLAG3* pMMC18 x MMC544	This work
MMC557	*parA*′*-FLAG3,* P_aTc_-*NgncR_093* pMMC38 x MMC547	This work
MMC558	*parB*′*-FLAG3*, P_aTc_-*NgncR_093* pMMC38 x MMC538	This work
MMC562	P_opaB_-SL-*lacZ*, P_aTc_-*NgncR_093* pMMC38 x MMC545	This work
MMC563	P_opaB_-∆SL-*lacZ*, P_aTc_-*NgncR_093* pMMC38 x MMC546	This work
MMC564	P_opaB_-SL1mut5*-lacZ* parA-lacZ mut3 gene block x MMC546	This work
MS11	Wild type *N. gonorrhoeae*	Swanson, 1972
KH655	∆SL*_parA_* pKH502 x MS11	This work
MR661	MS11 locked *pilE*, WT SL*_traH_* – *lacZ* at *iga/trpB*	Ramsey et al., 2015
MR664	MS11 locked *pilE*, SL_*traH-*ΔA_ – *lacZ* at *iga/trpB*	Ramsey et al., 2015
***E. coli* strains**		
*E. coli* TAM1	Used for cloning for all non-BACTH constructs. *mcrA* Δ(*mrr-hsdRMS-mcrBC*) Φ*80lacZ*ΔM15 *ΔlacX74 recA1 araD139* Δ(*ara-leu*)*7697 galU galK rpsL endA1 nupG*	Active Motif
*E. coli* 10-beta	Used for cloning in BACTH vectors. Δ(*ara-leu*) *7697 araD139 fhuA* Δ*lacX74 galK16 galE15 e14-* Φ*80*d*lacZ*Δ*M15 recA1 relA1 endA1 nupG rpsL* (Str^R^) *rph spoT1* Δ(*mrr-hsdRMS-mcrBC*)	New England Biolabs
BTH101	Used for BACTH assays. (F-, *cya-99*, *araD139*, *galE15*, *galK16*, *rpsL1* (*Str r*), *hsdR2*, *mcrA1*, *mcrB1*)	Euromedex

Gonococcal transformations with pMMC38 were re-streaked for screening on GCB agar plates containing 2 μg/ml chloramphenicol (Cm2). The fastest-growing colonies from Cm2 plates were re-streaked to GCB + Cm10 plates, from which single colonies were isolated for PCR screening and sequence confirmation.

Synthetic DNA gene blocks were used to transform GC directly by spot transformation and introduce new constructs by homologous recombination at the *iga*/*trpB* complementation locus. Gonococci transformed by gene blocks were re-streaked onto GCB + 40 μg/ml X-gal agar plates. For MMC545 and MMC564, white colonies were chosen for PCR screening and sequence confirmation. For MMC546, blue colonies were chosen.

Construction of BACTH constructs is described in the “BACTH assays” section, below.

### Real-Time PCR

RNA isolation and qRT-PCR were performed as described in (Ramsey et al., 2015), using SYBR green reagents. When comparing MS11 and KH655, RNA isolation was performed using TRIzol and the Zymo Direct-zol RNA Miniprep kit. DNase and cDNA preparation were unchanged. Quantitation was achieved by the ΔΔC_T_ method or with standard curves from MS11 genomic DNA, and Student’s *t* tests determined significance following previous studies (Applied Biosystems, 1997; Yuan et al., 2006). Primers are listed in Table 2.

### Western Blotting

Western blots were performed on PVDF membranes against the FLAG epitope, with the exception of Supplementary Figure S4 (described below). After protein transfer, membranes were blocked with 5% milk in 1X TBS + 0.1% Tween 20 (TBST). M2 Mouse α-FLAG primary antibody (Sigma Aldrich) was used at a concentration of 1:20,000 in TBST. Goat α-mouse secondary antibody (Santa Cruz Biotechnology) was also diluted 1:20,000 for use. Samples containing 6 μg protein were loaded per lane unless otherwise noted. Protein amounts were determined using the Bradford assay (Bio-Rad). All blots were visualized using the LI-COR Odyssey® Fc imaging system. For subcellular fractionation samples, α-CAT (Sigma) was used at 1:14,000 and α-SecY (Genscript) at 1:5,000. Horseradish peroxidase-conjugated secondary antibody mouse α-rabbit (Santa Cruz Biotechnology) was used at 1:20,000 dilution.

The western blot for the subcellular fractionation experiment shown in Supplementary Figure S4 used 4 μg protein per lane, and was transferred onto a nitrocellulose membrane. Blocking and primary antibodies were performed as above. LtgA was detected using 1:5,000 mouse monoclonal α-LtgA (final concentration ~0.17 μg/ml) primary antibody. 800CW goat α-mouse secondary antibody was used to detect ParA-FLAG3, ParB-FLAG3, and LtgA, 680RD goat α-rabbit secondary antibody was used to detect SecY and CAT.

### Metabolite Screening

A non-piliated variant of *N. gonorrhoeae* strain MMC545 was grown from freezer stocks on GCB plates overnight. Colonies were swabbed into cGCBL to start 3 ml cultures at OD_540_ = 0.25, and cultures were grown to mid-log phase (2 h). Cultures were diluted back to OD_540_ = 0.3 with cGCBL and aliquoted into the Biolog Phenotype Microarrays (PMs) with pipetting to resuspend the desiccated compounds of interest. We tested PMs 5, 8, 9, 10, 12, 13, 15, 16 (Biolog, #12141, 12,183, 12,161, 12,212, 12,213, 12,215, 12,216, respectively). We performed *in vivo* β-galactosidase assays by incubating these plates in the Biotek Synergy HT plate reader for 12 h at 37°C with agitation. OD_492_, OD_540_, and OD_660_ reads were taken every 30 min. According to Tang et al., normalized β-galactosidase activity was calculated as $\frac{OD_{630}indigo}{OD_{492}celldensity}=\frac{a\times OD_{492}-OD_{630}}{b\times OD_{630}-OD_{492}}$ , where *a* = 0.762, the correction factor for cell density and *b* = 0.267, the correction factor for indigo. To calculate the correction factor a, OD_492_ and OD_630_ were measured for non-piliated MMC545 gonococcal cultures during 16.5 h growth in a blank Biolog plate (six wells, *n* = 204 data points). Plotting OD_630_ as a function of OD_492_ yielded a linear relationship with *R* = 0.977, and the slope of the linear line of best fit is the correction factor *a* (Tang et al., 2013).

### Disk Diffusion

GCB agar plates of piliated MMC545 were grown 16–20 h at 37°C, 5% CO_2_, then swabbed into 2–4 ml cGCBL. Dilutions of 10^−4^–10^−5^ (80 μl) were spread plated on GCB + 40 μg ml^−1^ X-gal plates. Atop the spread culture, a 0.25-inch disk (Hardy Diagnostics) was placed and saturated with 10 μl of the compound of interest. Colony color was assessed after 36–48 h of incubation at 37°C with 5% CO_2_ and colony color was visually assessed 36–48 h later.

### BACTH Assays

GGI genes were PCR amplified from MS11 chromosomal DNA using primers specified in Table 2. PCR products and vectors were restriction enzyme digested (specified in Table 2, “Enzyme” column) and ligated. BACTH vectors are specified in Table 1. Final plasmids were confirmed by DNA sequencing. Plasmids of interest were co-transformed into *E. coli* BTH101 and plated on LB agar plates with 0.5 mM IPTG, 40 μg/ml Xgal, and appropriate antibiotic selection (Table 1, antibiotic selection needed for both co-transformed plasmids). Plates were incubated 40–48 h at 30°C before assessing blue colony color. Antibiotics were used at the following concentrations: 100 μg/ml ampicillin, 50 μg/ml kanamycin. For β-galactosidase assays using BACTH constructs, cells were grown overnight at 30°C in LB with appropriate antibiotics and 0.5 mM IPTG and β-galactosidase activities were measured as described by Miller (1972).

### β-Galactosidase Assays

*Neisseria gonorrhoeae* assays were performed according to Ramsey et al. (2015). Briefly, *N. gonorrhoeae* was grown overnight on GCB plates and swabbed into cGCBL at an OD_540_ ~ 0.25. After 3 h of aerated growth by rotation, 0.5 ml samples were collected for protein quantification. Cultures were chilled for 20 min on ice, then cells were collected from 2 ml samples by centrifugation, resuspended in 400 μl Z buffer (Miller, 1972) containing 0.002% SDS (Ramsey et al., 2015), aliquoted into 96 well plates, and exposed to ONPG at final concentration of 0.92 mg/ml. Protein concentration was assessed by Bradford assay and substituted for optical density to calculate the output in Miller units (Miller, 1972). Absorbance measurements were taken using a BioTek Synergy HT plate reader. For *E. coli* carrying pAKK128 or pAKK129, overnight cultures were diluted to an OD_600_ of 0.25 in LB with 25 μg/ml chloramphenicol and grown at 37°C for 3 h with rotation. The OD_600_ of the cultures was measured. The cultures were placed on ice for 20 min, and then 1 ml was pelleted and the cells resuspended in 1 ml of Z buffer. A 10 μl volume of the cell suspension was mixed with 990 μl of Z buffer, then 40 μl of chloroform was added, and the samples were vortexed. Samples were incubated at 28°C for 5 min. Three 100 μl aliquots of each sample were placed in a flat bottom 96-well plate. A volume of 30 μl of ONPG (4 mg/ml) was added to each well, and the OD_420_ and OD_550_ were measured every 5 min. β-gal units were calculated using the Miller equation.

### Subcellular Fractionation

Isolation of soluble and total membrane fractions was performed according to Ramsey et al. (2014) with the following modifications: at least four 3 ml cultures of each strain were grown for each fractionation experiment. Washed cell pellets were resuspended in 0.5 ml 0.01 M Tris–HCl (pH 7.0) before sonication. Samples were sonicated for a total of 50-, 10-s intervals with ≥30 s on ice between each pulse. Ultracentrifugation was performed at 65,000 rpm in a Beckman TLA110 rotor for 1.5 h.

Outer membrane samples were also prepared as described in (Ramsey et al., 2014), although for this study cells were harvested from six gonococcal cultures, 4 ml each, in cGCBL grown from OD_540_ = 0.25 for 3 h. Cells were collected by centrifugation at 10,000 rpm for 10 min at 4°C and washed once with cold PBS before proceeding.

## Results

### Stem-Loop Structure Dictates Protein Expression of ParA and ParB

Investigations of the expression of the gonococcal T4SS have begun to reveal a complex regulatory network, with transcriptional, translational, and post-translational mechanisms all at play (Pachulec et al., 2014; Ramsey et al., 2014, 2015; Callaghan et al., 2021). Quantitative transcript data indicate that for both the *traH* operon (*traH*, *traG*, and *atlA*) and the *parA* operon (containing *parA* and *parB*), transcripts are readily detected *in vitro.* However, proteins encoded on the *traH* operon are difficult to detect and attempts to visualize ParA and ParB expression have yet to be reported (Ramsey et al., 2015). The expression of TraH and TraG was shown to be controlled by an RNA switch, and we report here that *parA* uses a similar switch. We discovered a putative pair of stem-loops in the 5′ UTR of *parA*, by manual curation of intergenic GGI regions. The stem-loop proximal to the promoter (“SL2”) occludes the translational start site (TSS) and Shine-Dalgarno sequence of the *parA* operon mRNA (Figures 1A,B). We were unable to identify an energetically favorable alternate secondary structure that releases any part of the ribosome binding site (RBS) in the *parAB* 5′UTR secondary structure.

To determine the necessity of the stem-loop structure for regulation, we deleted the inner portion of the stem-loop sequence. By removing the inside “leg” of each stem (legs B and C, creating “SL_ΔBC_”) the formation of the secondary structure becomes much less favorable, increasing the Gibbs free energy (ΔG) of the structure from −25.3 to −4.68 kcal/mol (Figure 1B). This deletion also removes the bases that pair with the TSS “AUG” in the wild-type structure, leaving it more easily accessible. We cloned the wild-type and SL_ΔBC_ 5′UTR*_parA_* constructs into *E. coli* plasmids, making translational fusions with a *lacZ* reporter. The fusions were made such that the *lacZ* start codon and subsequent coding sequence replaced those of *parA*. The wild-type 5′UTR resulted in low levels of LacZ activity, whereas the SL_ΔBC_ mutant gave approximately 10-fold increased levels (Figure 1C). These data indicate that the stem-loop structures are functional in gene regulation and can perform such regulation in the absence of gonococcal-specific factors.

**Figure 2:**
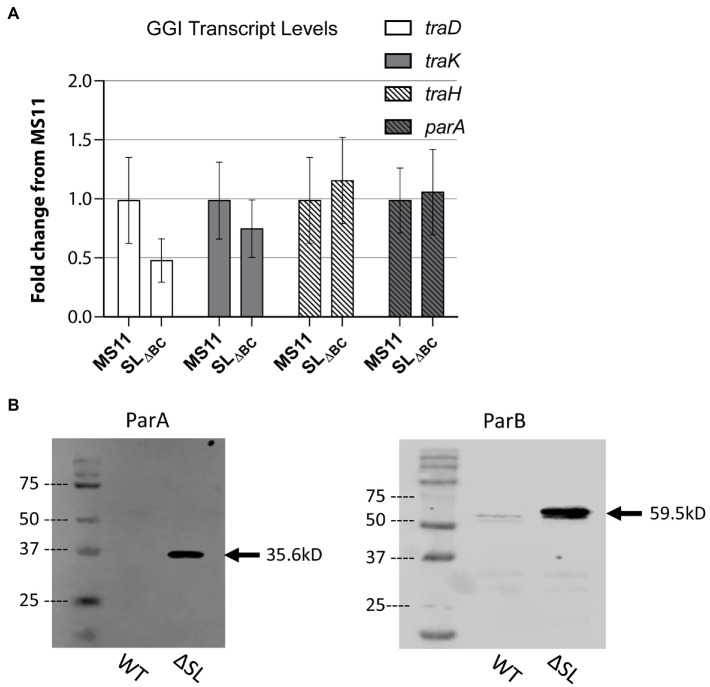
Stem-loop structure controls ParAB expression. **(A)** qRT-PCR measuring GGI transcript levels for T4SS genes in wild-type *Neisseria gonorrhoeae* strain MS11 and SL_∆BC_ mutant (KH655). Data shown are three replicates normalized to *rpoB*. Error bars are 95% confidence intervals. No significant differences by Student’s *t* test comparing ΔC_T_ values (*p* = 0.48, 0.72, 0.88, and 0.93 for *traD*, *traK*, *traH*, and *parA*, respectively). **(B)** Western blots of ParA-FLAG3 and ParB-FLAG3 comparing expression in wild type and SL_∆BC_ expression. Arrow indicates the expected band size.

We introduced the SL_ΔBC_ mutation into *N. gonorrhoeae* and examined effects on transcription and translation. We measured relative transcript abundance using qRT-PCR to test for transcriptional effects, looking for direct effects on *parA* or possible indirect effects on other T4SS genes. The SL_ΔBC_ deletion did not significantly alter transcript levels for any of the four tested genes, one gene from each of the four GGI operons necessary for secretion (operon 1: *traD*, operon 2: *traK*, operon 3: *traH*, terminal operon: *parA*; Figure 2A). This result suggests that the secondary structure is not a determinant of transcription activity nor mRNA stability for *parA*, nor does it affect transcript levels for genes in other T4SS operons.

Since SL2 would occlude the ribosome binding site and start codon of the *parA* mRNA, we next asked whether the stem-loops control protein expression. To detect the partitioning proteins by western blot, we added a FLAG3 epitope tag (three repeat copies of the FLAG epitope tag in tandem) to the C-terminus of either ParA or ParB by making genetic changes at the native loci. The epitope-tagged constructs were introduced into both wild-type gonococci (MS11) and the stem-loop deletion strain. In the wild-type background, ParA-FLAG3 was undetectable by western blot, and ParB-FLAG3 was very faintly visible. However, in the stem-loop mutant strains, expression of both proteins was greatly increased and easily visualized *via* western blotting against the FLAG epitope (Figure 2B). The control of ParB translation by a switch regulating ParA expression is possible because the start codon of *parB* overlaps the stop codon of *parA*, making it likely that *parA* and *parB* are translationally coupled like many of the gonococcal T4SS genes (Hamilton et al., 2005). We conclude that the stem-loops in the *parA* 5′UTR control protein expression from the *parA-parB* mRNA, revealing a putative riboswitch mechanism of control.

### Stem-Loop 1 Contributes to Riboswitch Architecture

**Figure 3:**
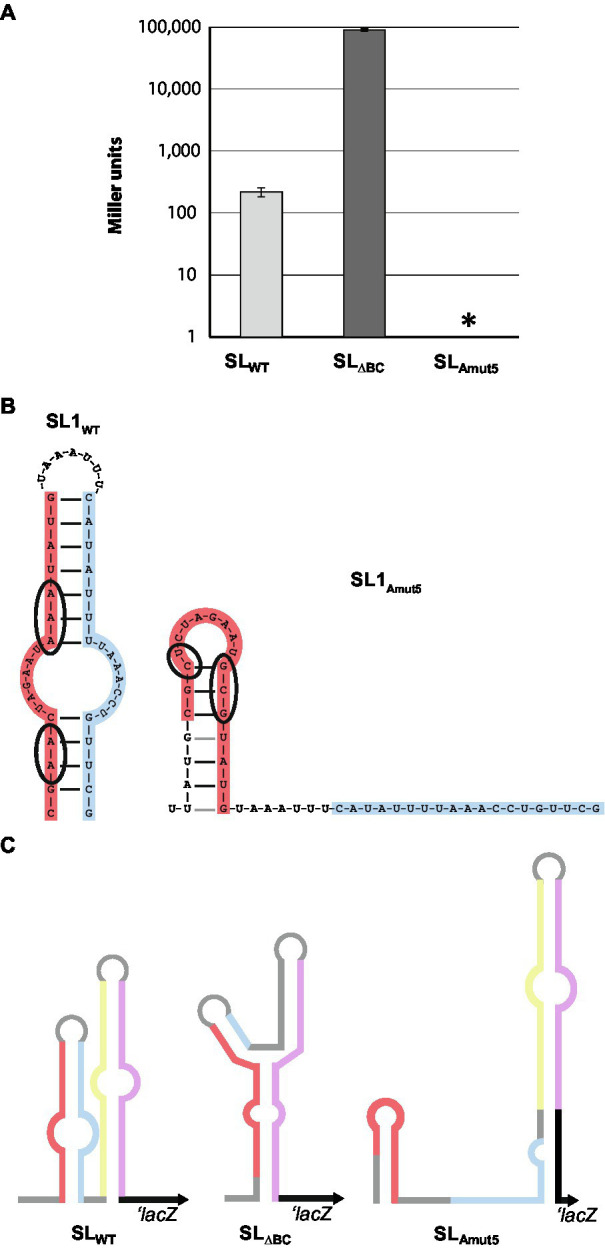
Genetic manipulation to up- and down-regulate protein expression. **(A)** Five base pair changes introduced to SL1 (left) lead to altered predicted secondary structure of the stem-loop region (right). Mutated bases are circled in wild-type and mutated SL1 diagram. **(B)** β-galactosidase assays of wild-type, SL_∆BC_, and SL_Amut5_ LacZ reporters. Data shown is averaged from three separate experiments. Note that the y-axis is in log scale. Error bars are SDs. ^*^p < 0.05 by Student’s *t* test compared to SL_WT_ (SL_ΔBC_: *p* = 0.14; SL_Amut5_: *p* = 0.00085). **(C)** Schematic of LacZ reporter constructs. Note that *lacZ* is involved in pairing with SL_Amut5_, as *parA* is also predicted to do.

For screening and semi-quantitative assessment of protein expression in *N. gonorrhoeae*, we introduced stem-loop – LacZ reporter constructs into the *iga*/*trpB* complementation locus on the gonococcal chromosome. We fused the *lacZ* gene to either the wild-type stem-loops (MMC545) or the stem-loop deletion sequence SL_ΔBC_ (MMC546) such that the *lacZ* translational start site is the native *parA* start site, normally occluded by the wild-type stem-loop structure. This construct was placed under the control of the *opaB* promoter, which is constitutively active in gonococci. β-galactosidase assays with these strains confirmed that the wild-type stem-loops expressed little LacZ protein whereas the stem-loop deletion mutant allows ample LacZ expression, increasing LacZ expression approximately 400-fold (Figure 3A).

Since no alternate structure for the 5′-UTR was identified, and the translation start site for ParA lies entirely on SL2 leg D, we questioned whether SL1 was playing a role in stem-loop-mediated regulation. To probe the utility of SL 1 in this system, we created a LacZ reporter strain with five base pair changes in SL1 leg A (SL_Amut5_), predicted to make folding of stem-loop 1 less favorable (ΔG_SL1-WT_ = −8.3 kcal/mol, ΔG_SL1-Amut5_ = −2.8 kcal/mol; Figure 3B). Surprisingly, these mutations abolished β-galactosidase activity to undetectable levels, below wild-type levels (Figure 3A), indicating a role for SL1 in structure and/or stability of the RNA secondary structure.

Sequence predictions of the mRNA containing SL_Amut5_ indicate a propensity for SL2 to elongate in the absence of strong SL1 folding (Figure 3C). At its native locus, a portion of SL1 leg B is able to pair with the beginning of the *parA* gene, creating six new base pairings and extending SL2. In the *lacZ* reporter constructs, SL2 is also predicted to elongate by pairing bases of SL1 leg B with the beginning of the *lacZ*, forming seven new base pairings in a slightly different configuration (Figure 3C). The predicted secondary structures of SL2-*parA* and SL2-*lacZ* are very similar, with ΔG = −18.5 and −19.1, respectively. The elongated SL2 structure has a more favorable free energy of folding (predicted ΔG_SL2_ decreases by 4.7 kcal/mol in the *lacZ* constructs, 2.8 kcal/mol in the *parA* constructs when it adopts the elongated conformation), which could explain the decreased protein output from the SL_Amut5_ construct.

Thus, it seems plausible that SL1 contributes to the formation of the wild-type SL2, and prevents the extension of SL2 into a longer and more stable structure. The wild-type stem-loop structure allows for a limited amount of protein expression – far lower than we observe in the complete disruption of these structures, but still detectable by β-galactosidase assay (Figure 3A). However, the formation of a structure with an even tighter occlusion of the ribosome binding site, as we observe in the absence of proper SL1 folding, introduces the possibility that binding of an unknown element of SL1 leg A could be a mechanism to completely abolish protein expression of ParAB. These stem-loop mutation results suggest a protein regulation system that can be finely tuned, both increasing and decreasing translation as the cell responds to environmental stimuli.

### Identification of Candidate Activators for ParAB Expression in Gonococci

If the 5′UTR sequence is a switch, what are its biologically relevant activators? We saw two potential avenues for RNA switch activation. Firstly, ligand binding could induce conformational changes that make the RBS more accessible to the ribosome. Alternatively, but not exclusively, an sRNA could interact with the stem-loops to alter their structure and allow translation initiation.

#### The sRNA *NgncR_093* Does Not Affect the RNA Switch

An RNA-Seq analysis by Remmele et al. (2014) identified several sRNAs within the GGI (Remmele et al., 2014). One such sRNA, NgncR_093, overlaps most of the *parA* gene beginning at base 664 (of the total 898 bp of *parA*) and continues, antiparallel, to cover the promoter and stem-loop regions (Figure 1A). Based on the reported transcription start site of NgncR_093, we mutated the predicted promoter sequence in wild-type gonococci to change two of the critical −10 residues using site-directed mutagenesis (Supplementary Figure S1A). This mutation did not alter *parA* transcript levels (Supplementary Figure S1B). We introduced the same NgncR_093 promoter mutations into the ParA-FLAG3 native expression strain and performed western blotting against the FLAG epitope tag. ParA was not detected in either the wild type or the NgncR_093 promoter mutant strain (data not shown).

Next, we asked if overexpression of NgncR_093 might alter ParAB expression, hypothesizing that the sRNA may bind to and alter the stem-loop structure of the *parAB* mRNA. We expressed NgncR_093 from a distant locus under inducible control of the *lac* promoter in the stem-loop-*lacZ* gonococcal reporter strains. Expression of NgncR_093 did not affect β-galactosidase activity in either the wild-type or mutant stem-loop reporters (Supplementary Figure S1C). Although the presence of the sRNA did not affect ParAB expression, it is still possible that local NgncR_093 transcriptional activity influences the RNA switch.

#### Screen for Metabolite Activators

We used Biolog Phenotype MicroArrays (PMs) to do a high-throughput screen for compounds that might activate expression from the RNA switch in strain MMC545, where the *parA* transcript is constitutively expressed and a LacZ reporter has been fused to the stem-loops. Based on the normalized β-galactosidase activity detection protocol of Tang et al. (2013), untreated plates were used to determine the correction factor for cell density and measure normalized β-galactosidase activity in control strains. MMC545 was then grown in PMs, where it was exposed to a panel of over 700 different metabolites. Several compounds increased LacZ expression in this screen. We identified the 11 compounds that yielded the highest normalized β-galactosidase activity (Supplementary Figure S2) and pursued further testing with these compounds.

As a method of verification, disk diffusion with promising compounds was performed using the wild-type stem-loop LacZ reporter construct. The following compounds were tested in X-gal disk diffusion assays: 100 mM adenine (in DMSO), 100 mM histidine, 100 mM glycine, 500 mM sodium phosphate buffer pH 7.0, 500 mM EDTA, 100 mM CuSO_4_, 500 mM sodium sulfate, 60% v/v sodium lactate solution, 100 mM 6-mercaptopurine (in DMSO), 100 mM CrCl_3_, and 100 mM His-His dipeptide (H-His-His-OH trifluoroacetate salt, Bachem). Only copper sulfate (CuSO_4_) had any visible effect on colony color (data not shown). Although the magnitude of activation by copper seen in the Biolog assays or on plates is only moderate, this finding aligns with other instances of copper-dependent enhancement of T4SS protein expression, described in (Callaghan et al., 2021).

### The ParA and ParB Encoded on the GGI Are Not Homologous to Known Cognate Pairs of Partitioning Proteins

The specific roles or mechanisms of partitioning activity have not been extensively explored in the gonococcal T4SS. Although we have built hypotheses around findings in other systems, there is ample variation in how these proteins function (Atmakuri et al., 2007; Lutkenhaus, 2012; Gruber et al., 2016). We decided to begin characterizing these proteins by looking at sequence homology, interaction partners, and localization.

Interestingly, the ParA and ParB encoded on the GGI may not be cognate partners. ParA contains a conserved domain from the P-loop NTPase superfamily of proteins, which are found abundantly in protein and DNA localization roles (Pfam accession cl38936; Marchler-Bauer et al., 2017; El-Gebali et al., 2019; Supplementary Figure S3). ParAB cognate pairs are canonically found adjacently encoded, which is indeed the case for the GGI (Bignell and Thomas, 2001; Hamilton et al., 2005; Pachulec et al., 2014; Figure 1A). On the other hand, the gonococcal ParB contains a conserved domain from the ParB family protein of the *Pseudomonas fluorescens* Pf-5 genetic island-1 (PFGI_1) class of integrating conjugative elements (Pfam superfamily cl26723, family TIGR03764; Marchler-Bauer et al., 2017; El-Gebali et al., 2019; Supplementary Figure S3). The founding members of this protein family are not encoded in immediate proximity to a ParA partner (Klockgether et al., 2004; Paulsen et al., 2005), and of the 41 protein architectures in the CDART database, only five have an adjacent P-loop NTPase (Pfam cl38936) domain (Geer et al., 2002). Consistent with this finding, neither nucleotide alignment search nor translated nucleotide alignment searches using the basic local alignment search tool (BLAST) identified homology of the *N. gonorrhoeae parAB* gene region to sequence from any organism outside of the *Neisseriaceae* family in which both *parA* and *parB* homologues were present, although *parA* and *parB* are individually homologous to many genes within their respective families (Altschul et al., 1990). Thus, while each gonococcal protein is likely to fit the role for one half of a partitioning protein pair, it is unclear whether these two proteins work together as a cognate pair, nor whether the GGI-encoded *parA* and *parB* were evolutionarily acquired as a unit.

### ParA and ParB Interact With the Relaxase, TraI

**Figure 4:**
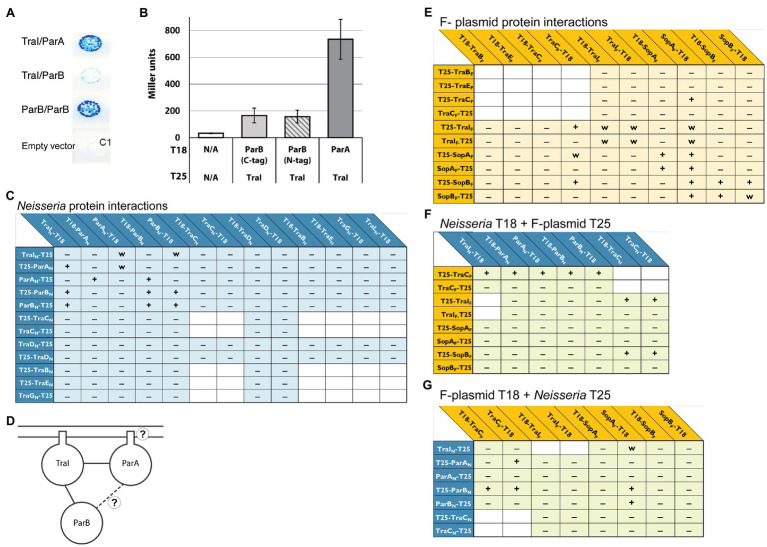
Bacterial two-hybrid interactions formed between *Neisseria* ParAB and TraI proteins. **(A)** Scanned image of an agar plate with colonies of *E. coli* BTH101 transformants carrying plasmids encoding the proteins indicated in the order T18/T25. C1 is an empty vector control: pUT18C/pKT25. **(B)** β-galactosidase assay testing expression from ParB-TraI and ParA-TraI interactions in BACTH constructs. Left to right: pUT18C/pKT25, pUT18TraI/pKT25ParB, pUT18TraI/p25NParB, pUT18TraI/pKT25ParA. Data shown are three replicates, error bars are SDs. **(C)** Interactions between *N. gonorrhoeae* ParA, ParB, and TraI with cytoplasmic and transmembrane proteins from *N. gonorrhoeae* T4SS. **(D)** Schematic drawing of all interactions identified between ParA, ParB, and TraI. **(E)** Interactions between F-plasmid homologues of the gonococcal T4SS. **(F,G)** Interactions between *Neisseria* and F-plasmid proteins. +, −, and w indicate interaction, no interaction, and weak interaction, respectively, tested in this study. Interactions tested in previous studies have been omitted. The placement of T18 and T25 relative to the protein name indicates N- or C-terminal fusion. T18 or T25 indicate that the gene encoding the protein was cloned into the BACTH vectors.

We used a Bacterial Two-Hybrid (BACTH) system to test for direct interactions between ParA and ParB with the other predicted cytoplasmic and transmembrane proteins of the gonococcal T4SS. This system uses two fragments, T18 and T25, of the catalytic domain of *Bortedella pertussis* adenylate cyclase, fused to the N- or C-terminal end of two proteins of interest. If an interaction between the proteins of interest brings T18 and T25 into sufficient proximity, functional complementation results in cAMP synthesis inducing transcriptional activation of the lactose operon (Karimova et al., 2001; Battesti and Bouveret, 2012). Using this system functional complementation can therefore be detected on agar plates with X-gal or by β-galactosidase assay. We tagged ParA and ParB with either T18 or T25 at both the N- and C-termini. These constructs were tested for interactions with the N- and C-termini of the other cytoplasmic and transmembrane gonococcal T4SS proteins: TraI, TraC, TraB, TraD, TraE, TraG, and TraL. Transmembrane proteins were tagged at the N- or C-terminus based on predicted topology, such that the tag will be cytosolic (Koch et al., 2020). This large screen identified only two definite interactions for each ParA and ParB: each protein gave a positive interaction result with itself and with TraI, the T4SS relaxase. Only one combination of fusion proteins indicated an interaction between ParA and ParB directly: ParA-T25 interacted with T18-ParB, but none of the other combinations gave a positive result (Figures 4A–C).

### Gonococcal Relaxosome Components Can Form Interactions With *E. coli* F-Plasmid Proteins

The plasmid partitioning proteins of F-plasmid, SopA and SopB, constitute a Walker-type ATPase (SopA) and DNA-binding partner (SopB; Watanabe et al., 1992; Schumacher and Funnell, 2005). We used the BACTH system to test for interactions between F-plasmid SopAB and TraI, looking to gain information on where the gonococcal system parallels or differs from better-characterized T4SSs. Additionally, we used this system to ask whether our gonococcal proteins of interest were able to interact with their counterparts in the F-plasmid system.

We created both N- and C-terminal fusions of SopA, SopB, and TraI from F-plasmid with the T18 and T25 fragments and tested them for interactions amongst themselves and with elements of the putative gonococcal relaxosome, as well as the cytoplasmic ATPase TraC (a homologue of VirB4, the most conserved element across T4SSs; Alvarez-Martinez and Christie, 2009; Guglielmini et al., 2013; Koch et al., 2020). For clarity, F-plasmid proteins will be specified by “F” (e.g., TraI_F_) and *Neisseria* proteins by “N” (e.g., TraI_N_) for these constructs. Apart from the expected dimerizations for the SopA_F_ and SopB_F_ proteins and the expected SopA_F_/SopB_F_ interaction (Bartosik et al., 2014), we observed a weak TraI_F_ dimerization and weak SopA_F_/TraI_F_ interactions (Figure 4D). For unknown reasons co-expression of a plasmid expressing TraC_F_ and a plasmid expressing ParB, TraI and in particular ParA homologues led to a decreased cell number in overnight cultures.

Interactions between F-plasmid partners helped confirm the utility of our approach and identified a parallel relaxase-partitioning protein interaction. Several mixed interactions have been reported between proteins of the F-plasmid and gonococcal systems previously, however none testing elements of the putative gonococcal relaxosome (Koch et al., 2020). The following cross-system interactions were observed, however in none of these cases were the proteins seen interacting in all possible N- and C-terminal or T18- and T25-terminal configurations: TraC_F_-ParA_N_ (3 of 8 potential interactions) and TraC_F_-ParB_N_ (4 of 8 potential interactions), SopA_F_-ParB_N_ (2 of 4 potential interactions), SopB_F_-TraC_N_ (2 of 4 potential interactions) and TraI_F_-TraC_N_ (2 of 4 potential interactions; Figures 4E,F).

### Subcellular Localization of ParA and ParB

**Figure 5:**
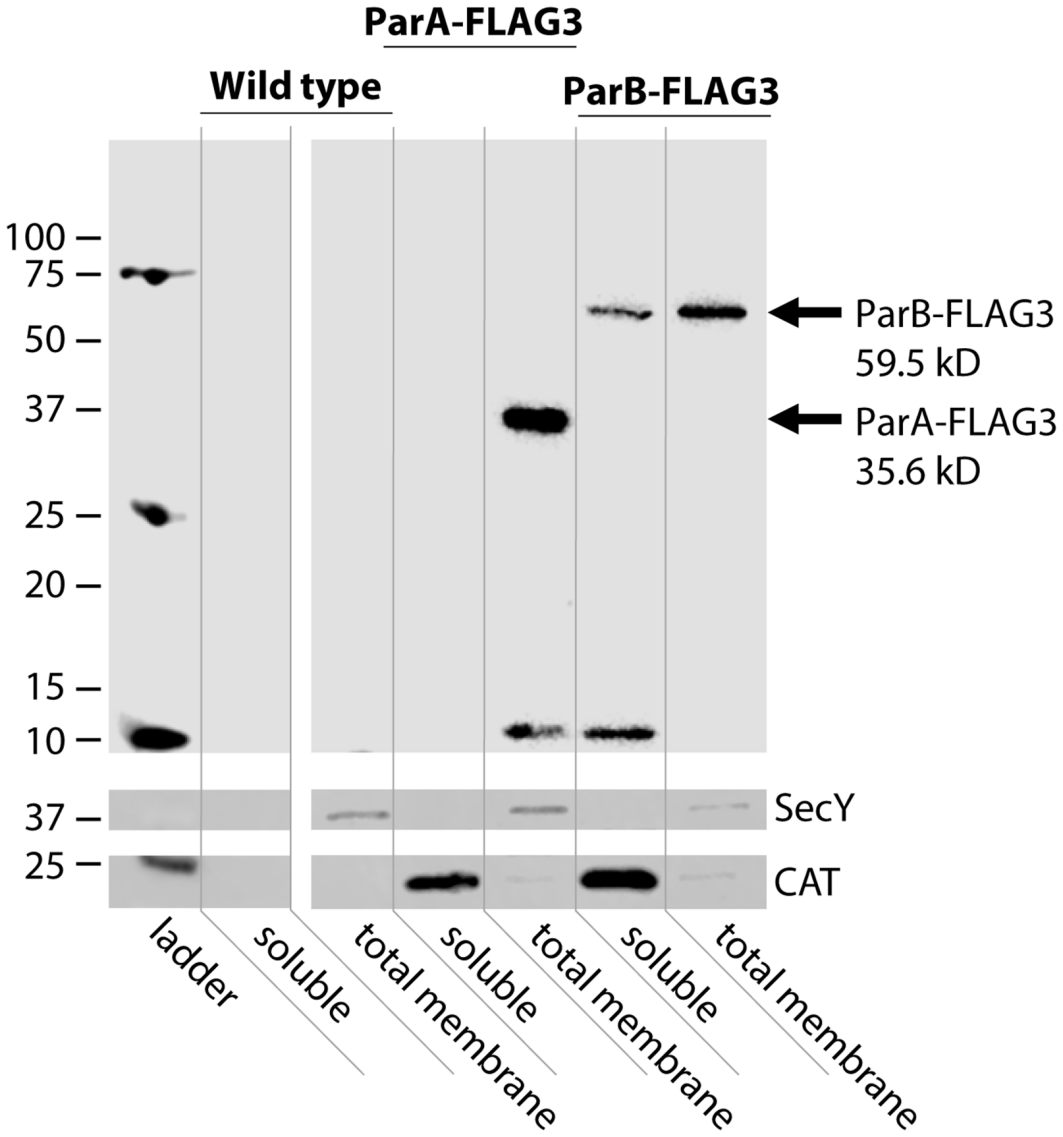
Subcellular fractionation of ParA and ParB. Western blot against the FLAG epitope to detect ParA-FLAG3 and ParB-FLAG3 expressed from the native locus in the SL_ΔBC_ strain background. The inner membrane protein SecY is a total membrane fraction control, CAT is a cytosolic control (Ramsey et al., 2014).

To better understand where the partitioning proteins act to facilitate DNA secretion, subcellular fractionation of FLAG3-tagged ParA and ParB was used to separate soluble from membrane-associated proteins. Strains used for the fractionation studies had the stem-loop deletion in the native site *parA* 5′UTR to overexpress ParAB and allow visualization on western blots. These strains also had the chloramphenicol acetyltransferase gene *cat* expressed at the *aspC*/*lctP* complementation site, to be used as a cytosolic protein control (Ramsey et al., 2014). Based on sequence predictions, we expected both proteins to be entirely cytosolic (Bernsel et al., 2009). However, western blotting against the FLAG epitope revealed that ParA fractionated exclusively with the membrane fraction of culture lysates. Furthermore, ParB is present in both the soluble and membrane fractions, with the membrane fraction having greater ParB signal than the soluble fraction (Figure 5). Isolation of outer membrane proteins from the total membrane fraction revealed no ParA or ParB in the outer membrane, indicating that both proteins associate with the inner membrane (Supplementary Figure S4).

## Discussion

The partitioning proteins ParA and ParB of the gonococcal T4SS are integral to ssDNA secretion. Canonically, partitioning proteins act in cognate pairs to accurately segregate chromosomes and/or plasmids. However, gonococcal ParA and ParB are not an obvious cognate pair; while they are encoded adjacent to one another on the same operon, their conserved domains exhibit homology to differing classes of partitioning proteins. We found limited evidence to support a direct ParA-ParB interaction. We did find evidence that both ParA and ParB interact with themselves and the relaxase TraI, supporting the existing hypothesis that a ParAB-TraI relaxosome facilitates DNA nicking during the initiation of secretion. These results suggest that ParA and ParB might function in a novel way, working to initiate secretion by associating with TraI without interacting with one another.

Fractionation experiments indicate an association of both partitioning proteins with the bacterial inner membrane. These results were surprising because the canonical action of partitioning proteins led us to expect that at least one of these proteins will associate with DNA in the cytosol. Sequence-based analysis using the SignalP 5.0 and TOPCONS algorithms predicted no probable transmembrane domains in either protein and a low likelihood signal peptide in ParA (Bernsel et al., 2009; Juan et al., 2019). Examination of the N-terminal region of ParA with the Helical Wheel generator program EMBOSS pepwheel^1^ suggests that amino acids 21–28 may form an amphipathic alpha-helix that could interact with the membrane.

**Figure 6:**
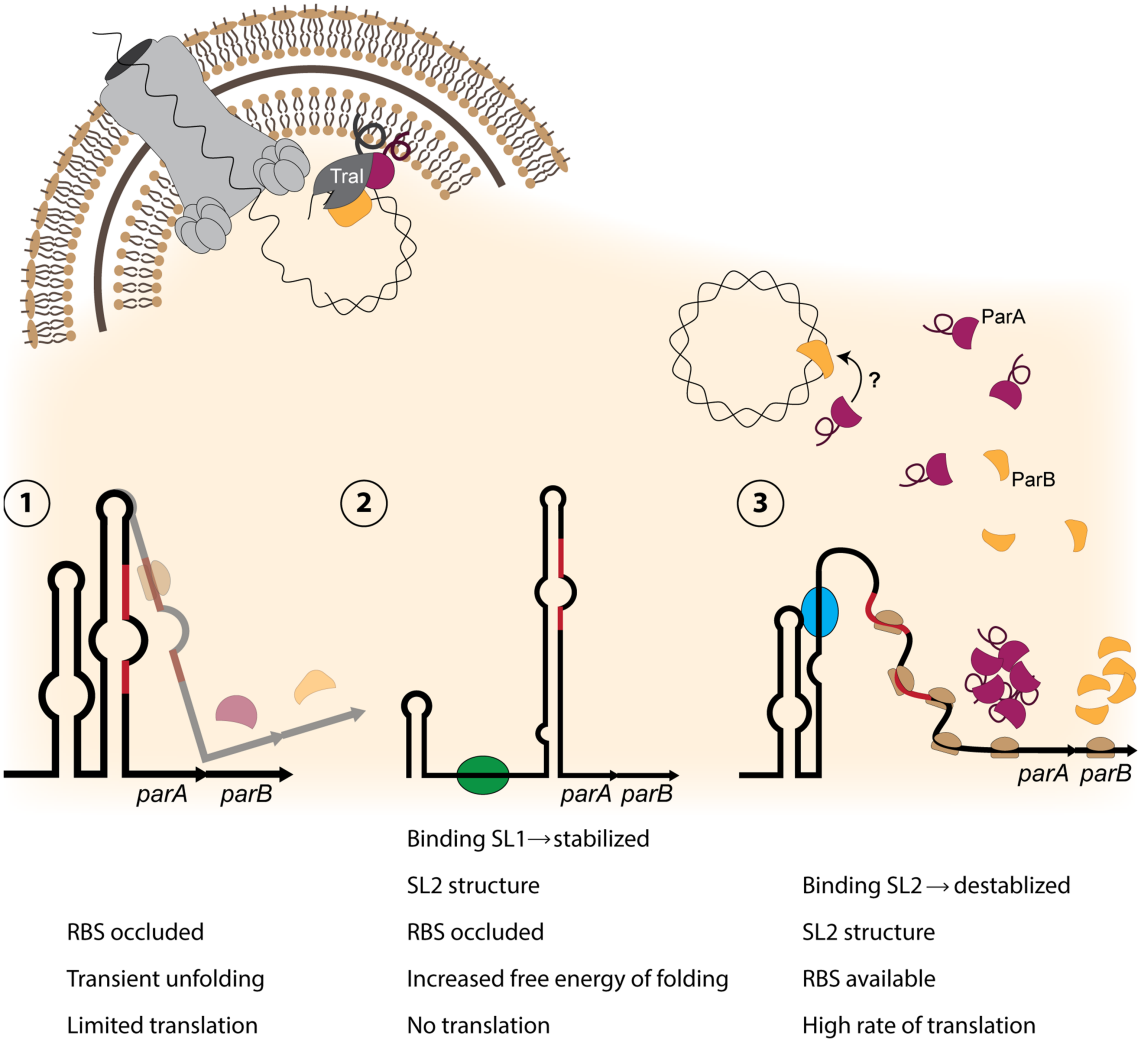
Model of partitioning protein activity in the gonococcal T4SS. (1) The *parAB* transcript contains an RNA-switch consisting of two stem-loops, with stem-loop 2 (SL2) occluding the Shine-Dalgarno sequence and the start codon (red regions) from binding the ribosome. Only a small amount of translation occurs. (2) If stem-loop 1 is destabilized, possibly by a protein or sRNA (green oval) binding to SL1 sequence, SL2 forms an extended structure, preventing translation. (3) If SL2 is destabilized by a factor (blue oval) binding within the SL2 sequence, a high rate of translation can occur. Production of ParA (burgundy) and ParB (yellow) allows for relaxosome formation with ParB binding chromosomal DNA (top right). It is possible that ParA binds ParB. ParA and TraI (dark gray) associate with the inner membrane through amphipathic alpha-helix regions (looped line), and ParB binds TraI. TraI nicks the DNA, and it may be transported into the medium through the T4SS apparatus (top left).

Our finding that ParA and ParB both interact with TraI provides an alternate explanation to membrane or transmembrane ParAB proteins; TraI associates with the inner membrane *via* an amphipathic helix and fractionates with cellular membranes (Salgado-Pabón et al., 2007). Disruption of this helix causes TraI to fractionate with the soluble proteins (Salgado-Pabón et al., 2007). Thus ParA and ParB might each bind to membrane-associated TraI, and the three proteins may form a relaxosome complex at the inner membrane (Figures 4B, 6).

If the entire relaxosome assembles at the inner membrane, we are left with new questions about substrate localization. How does the relaxosome recruit chromosomal DNA for nicking, and what caused this novel localization to develop in the gonococcal T4SS? Although the chromosome is cytosolic, perhaps transient association with the membrane is sufficient to allow interaction with a membrane-associated relaxosome. Alternatively, more aligned with other T4SS partitioning systems, the key may lie in the dual-localization of ParB in both the cytosol and membrane fraction. As the DNA-binding entity, we may speculate that the role of recruitment falls to ParB, which complexes the DNA to be nicked with our membrane-associated TraI, and (directly or indirectly) works in conjugation with ParA ATPase activity to initiate secretion (Figure 6).

Several instances of stem-loop-mediated regulation have been reported in the pathogenic *Neisseria* (Loh et al., 2013; Ramsey et al., 2015; Masters et al., 2016). We grow this body of literature by presenting a previously unknown RNA switch upstream of *parA* that contributes to the regulation of the gonococcal T4SS by controlling the expression of the partitioning proteins ParAB. The *parAB* switch consists of two stem-loops, which we have termed SL1 and SL2. Folding of SL2 occludes the Shine-Dalgarno sequence and the start codon of the *parA* mRNA. Complete disruption of both stem-loops greatly increases ParAB protein expression, whereas disruption of SL1 formation abolishes protein expression. We did not observe any effects from promoter disruption or ectopic overexpression of NgncR_093, the sRNA overlapping *parA* and the stem-loop region. Thus the function of this sRNA remains a mystery.

Stem-loop structure could be manipulated by a variety of mechanisms to effectively control protein expression. Since significant disruption of the secondary structure allows huge amounts of protein expression, a classic riboswitch mechanism in which ligand binding causes conformation change to allow expression seems likely. The potential to turn expression entirely “off” introduces more complexity and nuance to this system. Perhaps the folding of SL1 keeps the extended SL2 from becoming energetically favorable, maintaining low levels of ParAB expression (Figure 6). However, there may be other factors at play; stabilization of SL1 could act as a mechanism to allow or increase protein expression under certain conditions. Identification of regulatory elements here is challenging; because laboratory GGI expression is very different than in the human host – relevant ligands, sRNAs, and/or proteins may not be expressed *in vitro* (Callaghan et al., 2021).

Together, our data suggest that the *parAB* RNA switch can be finely tuned, allowing for precise control of ParAB expression at the translational level. We speculate that since ParAB activity in the relaxosome results in chromosomal nicking, and potentially initiates the ssDNA secretion process, the expression of these proteins needs to be tightly regulated to prevent unnecessary DNA damage by the relaxase and wasteful ATP-dependent secretion when it has no benefit to the bacterial cell or population. Additionally, extracellular DNA can elicit robust host immune responses, so careful regulation to avoid DNA secretion when evading the host immune system may be paramount to T4SS regulation (Hemmi et al., 2000).

A large-scale metabolite screen identified several compounds as potential activators of the RNA switch. Of these, we confirmed modest, concentration-dependent upregulation from copper sulfate. Copper has been shown to alter T4SS protein expression previously, and this activation was speculated to occur when gonococci are in the macrophage phagosome (Callaghan et al., 2021). This finding opens a line of inquiry regarding copper binding or indirect activation of the RNA switch. More extensive testing is required to fully characterize this newly reported regulatory element. Riboswitch ligands vary widely, including proteins, sRNAs, tRNAs, metals and metabolites. Temperature and pH-responsive riboswitches have also been described (Winkler and Breaker, 2005; Nechooshtan et al., 2009; Loh et al., 2013; Sherwood and Henkin, 2016).

The *parAB* stem-loop regulator is the second RNA switch identified on the GGI; there is also a stem-loop structure upstream of *traH* that can form an alternate fold to activate protein expression (Ramsey et al., 2015). The activator(s) of the *traH* switch has not yet been identified. The occurrence of two stem-loop-based regulatory mechanisms in the 59 kb space of the GGI raises specific questions about mechanisms of T4SS regulation, but also broader questions regarding the levels of regulation and interplay between regulatory mechanisms at different sites of the GGI.

## Data Availability Statement

The original contributions presented in the study are included in the article/Supplementary Material; further inquiries can be directed to the corresponding author.

## Author Contributions

MC, AK, and JD: conceptualization. BK, KH, and AK: methodology. MC, BK, KH, AK, and RS: investigation. MC: writing – original draft. JD, MC, BK, KH, AK, and NK: writing – review and editing. JD and NK: supervision and funding. All authors contributed to the article and approved the submitted version.

## Funding

This work was funded by NIH grant R01AI047958. BK and NK were funded by the EPSRC (EP/N031962/1) and a Royal Academy of Engineering Chair in Emerging Technologies award.

## Conflict of Interest

The authors declare that the research was conducted in the absence of any commercial or financial relationships that could be construed as a potential conflict of interest.

## Publisher’s Note

All claims expressed in this article are solely those of the authors and do not necessarily represent those of their affiliated organizations, or those of the publisher, the editors and the reviewers. Any product that may be evaluated in this article, or claim that may be made by its manufacturer, is not guaranteed or endorsed by the publisher.

## Footnotes

^1^ https://www.bioinformatics.nl/cgi-bin/emboss/pepwheel
