# Supplementary material for: Expression, Localization, and Protein Interactions of the Partitioning Proteins in the Gonococcal Type IV Secretion System
Source: Front Microbiol. 2021 Dec 16;12:784483. doi: 10.3389/fmicb.2021.784483 (PMC8716806; doi:10.3389/fmicb.2021.784483)
Supplement: Supplementary file 1 [file Data_Sheet_1.pdf]

Supplemental Material

A

Strain	-10 promoter sequence
Wild type	TACGCT
Promoter mutant	**GACGGA**

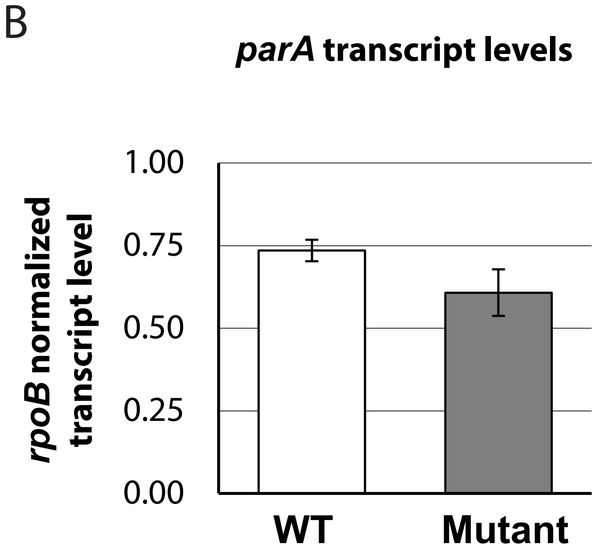

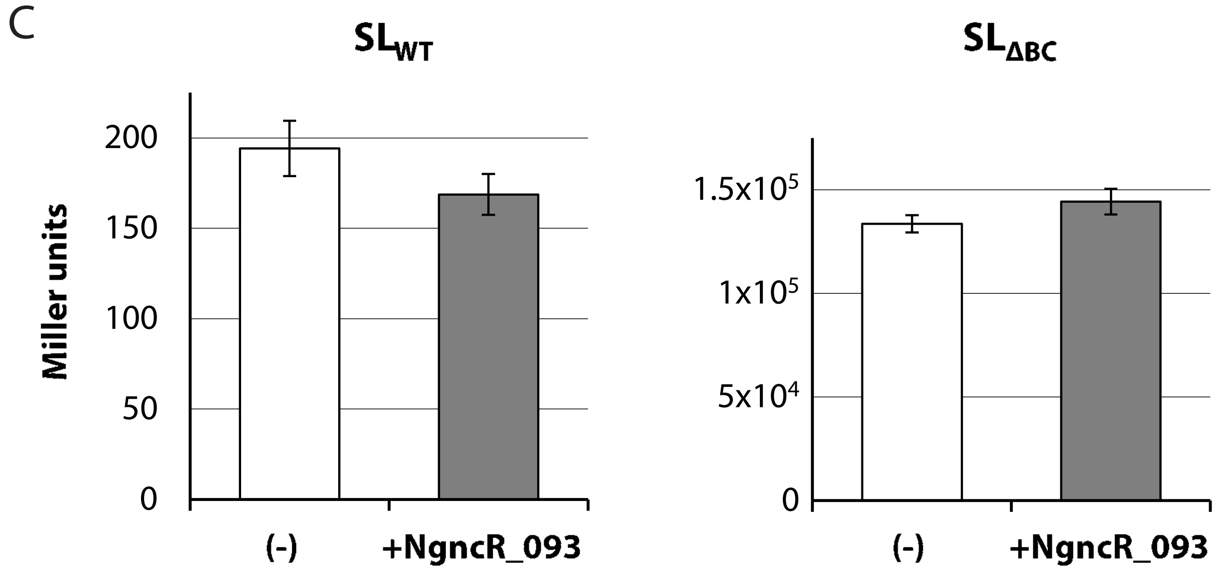

Figure S1. The sRNA *NgncR_093* does not show effects on transcript or protein levels. (A) Three base pair mutations were introduced to the -10 promoter element of P_{NgncR_093}. Mutated bases are depicted in bold font. (B) qRT-PCR shows *parA* levels are not altered by promoter mutation. "Mutant" indicates strain MMC544, where the 2 base pairs of the -10 element of the promoter sequence have been mutated. Data shown are two replicates, error bars are 95% confidence intervals. No significant difference by Student's t-test (p = 0.66). (C) β-galactosidase assay with SL_{WT} and $SL_{\Delta BC}$ LacZ reporters. Data shown are three replicates, error bars are standard deviations. Note the different axes between the two graphs. No significant differences by Student's t-test (p = 0.082, 0.069 for SL_{WT} and $SL_{\Delta BC}$, respectively).

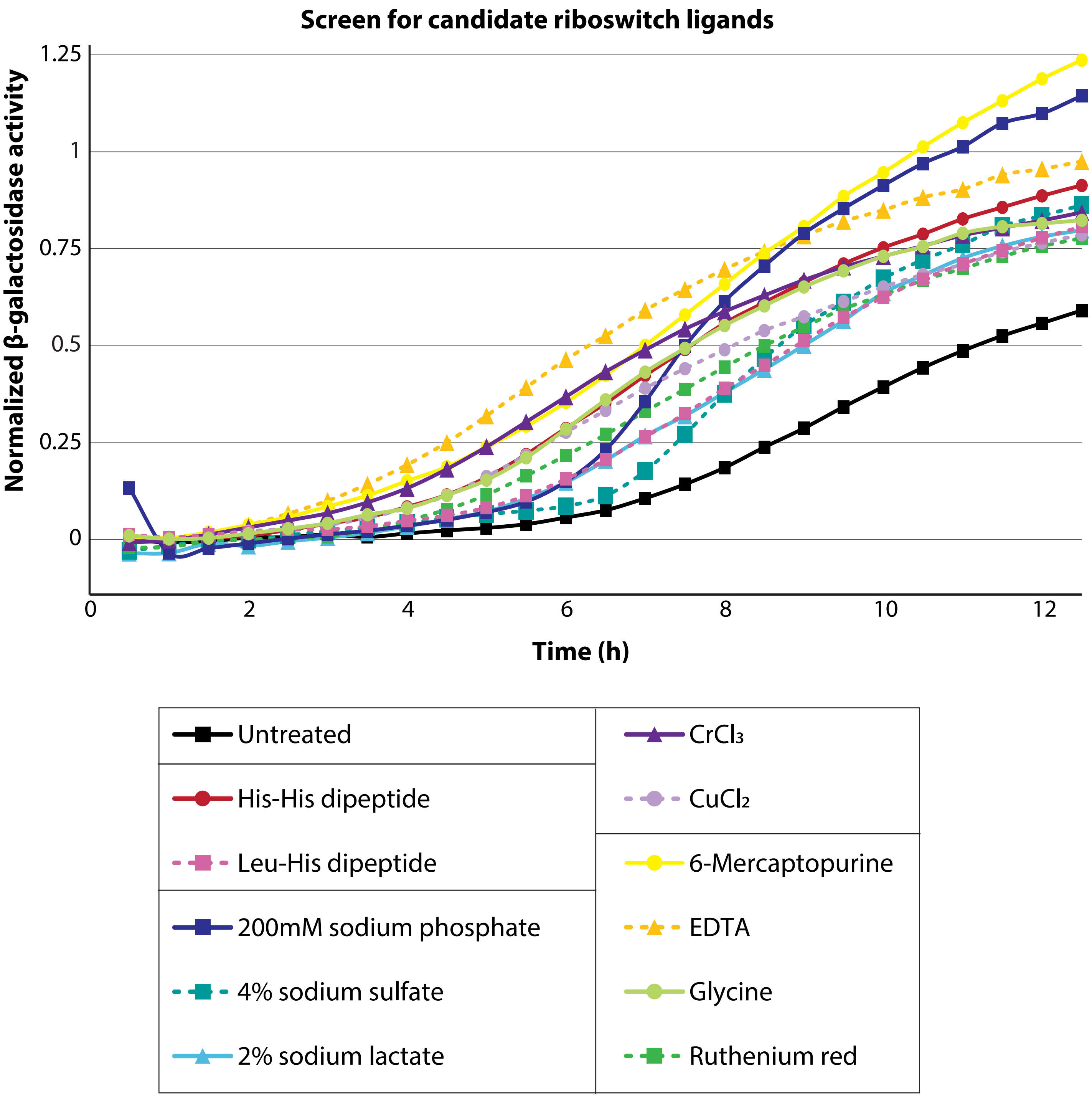

Figure S2. Candidate activators of the RNA switch. *N. gonorrhoeae* strain MMC545, carrying the wild-type *parA* stem-loops translationally fused to *lacZ*, was used to measure the effects of various compounds on translation. The reporter construct is transcribed at a high level in this strain from the constitutive *opaB* promoter. Compound screening yielded several candidates for putative riboswitch ligands.

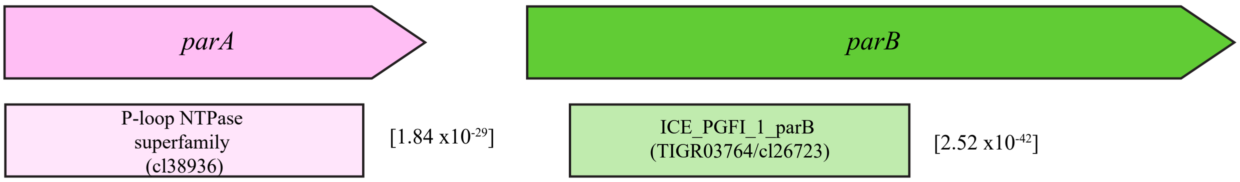

Figure S3. Alignments of GGI-encoded ParA and ParB with their most conserved protein domain. Conserved domains identified by CD-Search are presented as Name (Pfam accession ID). The E-value of each alignment is listed in brackets (Marchler-Bauer et al. 2017).

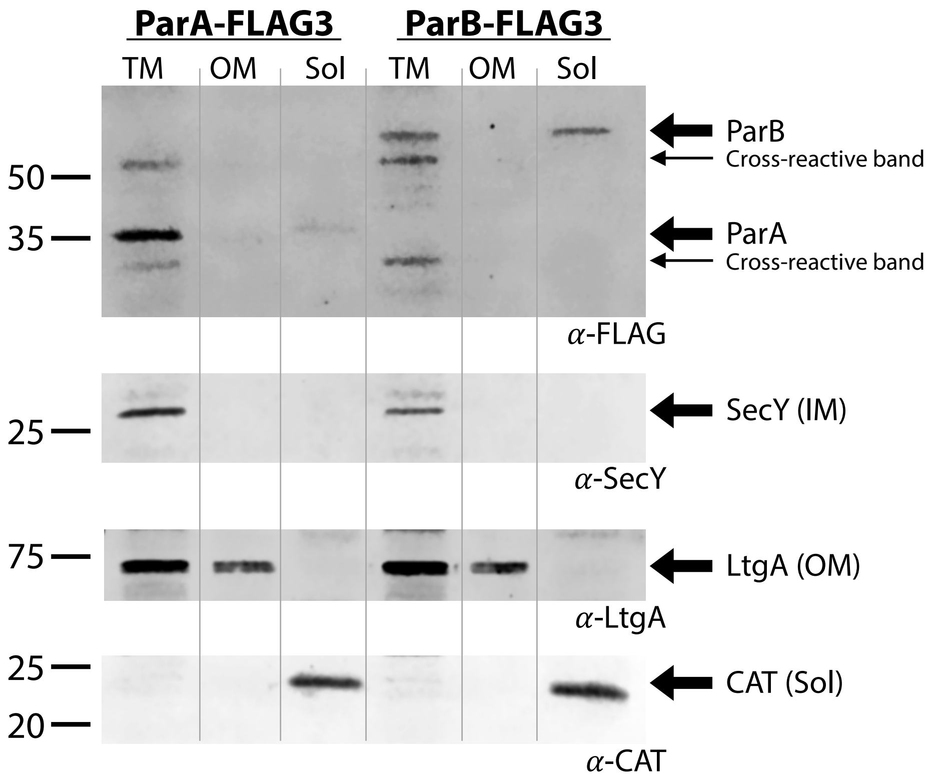

Figure S4. Western blotting against FLAG epitope-tagged ParA and ParB shows that these proteins are not found in the outer membrane. Results consistent with Figure 5 indicate that ParA is an inner membrane-associated protein, and that ParB is found both in association with the inner membrane and in the cytosol. A very faint ParA-sized band in the soluble fraction may reflect a small amount of soluble ParA as well. TM: total membrane; OM: outer membrane; Sol: soluble; IM: inner membrane. Fractions are listed across the top of the blot for each protein, controls are listed at the right. Ladder is reported in kDa. Primary antibody detected in each blot photo is indicated at the lower right of each photo.

Gene block sequences

parA-lacZ WT3 gene block
5′-
GCTCATGCCGTCTGAAACCGGAAAAGGCTTCAGACGGCATCAGTGTTAAAGTAAGTTCTATGGCAGTGAAAACACTA
ACGGCCGGACTGGATTTTGCTTATCGGTTTAAAGTCGGCAACCTTACCGTAAAACCCTTGTTATCTGCAGCTTACTT
TGCCAATTATGGCAAAGGCGGCGTGAATGTGGGCGGTAAATCCTTCGCCTATAAAGCAGATAATCAACAGCAATATT
CAGCAGGCGCCGCGTTACTGTACCGTAATGTTACATTAAACGTAAATGGCAGTATTACAAAAGGAAAACAATTGGAA
AAACAAAAATCCGGACAAATTAAAATACAGATTCGTTTCTAAAATACTAAATTTAAGGGATGCATAAACTGCATCCC
TTAAGGACTAGTCGGGCCCCCCCTCGAGCCATATTGTGTTGAAACACCGCCCGGAACCCGATATAATAAGATGTCGA
TTATCTTCGAACTAGAATAAATATGTAAATTTCATATTTTAAACCTGTTCGATTGCGGATGTTTTTCTCTTTATAAA
TCAATTAAATATAAGGAAATATTATGTCCGCAATTACGGATTCACTGGCCGTCGTTTTACAACGTCGTGACTGGGAA
AACCCTGGCGTTACCCAACTTAATCGCCTTGCAGCACATCCCCCTTTCGCCAGCTGGCGTAATAGCGAAGAGGCCCG
CACCGATCGCCCTTCCCAACAGTTGCGCAGCCTGAATGGCGAATGGCGCTTTGCCTGGTTTCCGGCACCAGAAGCGG
TGCCGGAAAGCTGGCTGGAGTGCGATCTTCCTGAGGCCGATACTGTCGTCGTCCCCTCAAACTGGCAGATGCACGGT
TACGATGCGCCCATCTACACCAACGTGACCTATCCCATTACGGTCAATCCGCCGTTTGTTCCCACGGAGAATCCGAC
GGGTTGTTACTCGCTCACATTTAATGTTGATGAAAGCTGGCTACAGGAAGGCCAGACGCGAATTATTTTTGATGGC
-3′

parA-lacZ mut2 gene block
5′-
GCTCATGCCGTCTGAAACCGGAAAAGGCTTCAGACGGCATTGTCATCTGCAGATTACAAGTTGGGTGACGACAGTGT
TAAAGTAAGTTCTATGGCAGTGAAAACACTAACGGCCGGACTGGATTTTGCTTATCGGTTTAAAGTCGGCAACCTTA
CCGTAAAACCCTTGTTATCTGCAGCTTACTTTGCCAATTATGGCAAAGGCGGCGTGAATGTGGGCGGTAAATCCTTC
GCCTATAAAGCAGATAATCAACAGCAATATTCAGCAGGCGCCGCGTTACTGTACCGTAATGTTACATTAAACGTAAA
TGGCAGTATTACAAAAGGAAAACAATTGGAAAAACAAAAATCCGGACAAATTAAAATACAGATTCGTTTCTAAAATA
CTAAATTTAAGGGATGCATAAACTGCATCCCTTAAGGACTAGTCGGGCCCCCCCTCGAGCCATATTGTGTTGAAACA
CCGCCCGGAACCCGATATAATAAGATGTCGATTATCTTCGAACTAGAATAAATATGTAAATTTCATAGGGTCATATG
TCAATTAAATATAAGGAAATATTATGTCCGCAATTACGGATTCACTGGCCGTCGTTTTACAACGTCGTGACTGGGAA
AACCCTGGCGTTACCCAACTTAATCGCCTTGCAGCACATCCCCCTTTCGCCAGCTGGCGTAATAGCGAAGAGGCCCG
CACCGATCGCCCTTCCCAACAGTTGCGCAGCCTGAATGGCGAATGGCGCTTTGCCTGGTTTCCGGCACCAGAAGCGG
TGCCGGAAAGCTGGCTGGAGTGCGATCTTCCTGAGGCCGATACTGTCGTCGTCCCCTCAAACTGGCAGATGCACGGT
TACGATGCGCCCATCTACACCAACGTGACCTATCCCATTACGGTCAATCCGCCGTTTGTTCCCACGGAGAATCCGAC
GGGTTGTTACTCGCTCACATTTAATGTTGATGAAAGCTGGCTACAGGAAGGCCAGACGCGAATTATTTTTGATGGC
-3′

parA-lacZ mut3 gene block
5′-
GCTCATGCCGTCTGAAACCGGAAAAGGCTTCAGACGGCATCAGTGTTAAAGTAAGTTCTATGGCAGTGAAAACACTA
ACGGCCGGACTGGATTTTGCTTATCGGTTTAAAGTCGGCAACCTTACCGTAAAACCCTTGTTATCTGCAGCTTACTT
TGCCAATTATGGCAAAGGCGGCGTGAATGTGGGCGGTAAATCCTTCGCCTATAAAGCAGATAATCAACAGCAATATT
CAGCAGGCGCCGCGTTACTGTACCGTAATGTTACATTAAACGTAAATGGCAGTATTACAAAAGGAAAACAATTGGAA
AAACAAAAATCCGGACAAATTAAAATACAGATTCGTTTCTAAAATACTAAATTTAAGGGATGCATAAACTGCATCCC
TTAAGGACATGTCGGGCCCCCCCTCGAGCCATATTGTGTTGAAACACCGCCCGGAACCCGATATAATAAGATGTCGA
TTATCTTCGCTCTAGAATGCGTATGTAAATTTCATATTTTAAACCTGTTCGATTGCGGATGTTTTTCTCTTTATAAA
TCAATTAAATATAAGGAAATATTATGTCCGCAATTACGGATTCACTGGCCGTCGTTTTACAACGTCGTGACTGGGAA
AACCCTGGCGTTACCCAACTTAATCGCCTTGCAGCACATCCCCCTTTCGCCAGCTGGCGTAATAGCGAAGAGGCCCG
CACCGATCGCCCTTCCCAACAGTTGCGCAGCCTGAATGGCGAATGGCGCTTTGCCTGGTTTCCGGCACCAGAAGCGG
TGCCGGAAAGCTGGCTGGAGTGCGATCTTCCTGAGGCCGATACTGTCGTCGTCCCCTCAAACTGGCAGATGCACGGT
TACGATGCGCCCATCTACACCAACGTGACCTATCCCATTACGGTCAATCCGCCGTTTGTTCCCACGGAGAATCCGAC
GGGTTGTTACTCGCTCACATTTAATGTTGATGAAAGCTGGCTACAGGAAGGCCAGACGCGAATTATTTTTGATGGC
-3′
